# Blastoid Variant Mantle Cell Lymphoma Expressing Aberrant CD3 and CD10 with Concurrent Small Lymphocytic Lymphoma: Establishment of a Clonal Relationship by B- and T-Cell Receptor Gene Rearrangements

**DOI:** 10.1155/2018/8303571

**Published:** 2018-12-05

**Authors:** Christopher L. Felten, Joel A. Chan, Dulce R. DeCastro, Jean Lopategui, Swapnil P. Rajurkar

**Affiliations:** ^1^Gemini Diagnostics Hematopathology, 756 Lakefield Road Suite C, Westlake Village, CA 91361, USA; ^2^San Antonio Regional Hospital, Department of Pathology, 999 San Bernardino Road, Upland, CA 91786, USA; ^3^Cedars-Sinai Medical Center, Department of Pathology, 8700 Beverly Boulevard, Los Angeles, CA 90048, USA; ^4^Medical Oncology and Hematology, 7777 Milliken Avenue Suite 210, Rancho Cucamonga, CA 91730, USA

## Abstract

Mantle cell lymphoma (MCL) is an aggressive non-Hodgkin B-cell lymphoma typically expressing CD19, CD20, CD5, FMC-7, CyclinD1, and SOX-11 and harboring the IgH/CCND1 translocation. We report a blastoid variant of mantle cell lymphoma (MCL) involving an inguinal lymph node that, in addition to classical phenotypic and genetic findings, also aberrantly coexpresses surface CD10 and cytoplasmic CD3. Small lymphocytic lymphoma (SLL) was also present in the same lymph node and in the bone marrow. B- and T-cell gene rearrangement studies by PCR show the MCL and SLL to be clonally related. Expression of multiple aberrant antigens and concurrent lymphomas of different classifications can cause a diagnostic challenge. Awareness of such a presentation and integration of the data from morphologic evaluation, flow cytometry, immunohistochemistry, and FISH studies is required for proper diagnosis, prognosis, and therapy.

## 1. Introduction

Classical mantle cell lymphoma (MCL) is a neoplasm typically of monomorphic small- to medium-sized cells with slight to marked irregular nuclear contours growing in a diffuse or nodular architecture. It does not contain transformed cells resembling centroblasts, immunoblasts, or paraimmunoblasts [1]. By flow cytometric phenotyping, it typically expresses pan B-cell markers (CD19 and CD20), the T-cell antigen CD5, and FMC-7 while lacking CD23, CD11c, and CD10 [2–4]. Immunohistochemistry further shows that it expresses CyclinD1 (BCL-1) and SOX-11 [5, 6]. In addition, the genetic hallmark of the disease, a t(11;14)(q13;q32)–IgH/CCND1 translocation, can be identified by FISH or molecular studies [7].

Herein, we report a unique case of blastoid variant of mantle cell lymphoma that, in addition to the typical expression of CD20, CD5, FMC-7, CyclinD1 (BCL-1), and SOX-11 also showed aberrant positivity for CD10 and cytoplasmic CD3. The diagnosis was further complicated by the additional presence of a minor small lymphocytic lymphoma (SLL) component within the same biopsy.

## 2. Case Presentation

A 72-year-old male presented with a left groin lump which grew steadily over the course of several months. He denied any fever, chills, night sweats, or weight loss. CT scan revealed a 5.3 cm left inguinal mass, highly suspicious for lymphoma. No other lymphadenopathy was identified in the abdomen or pelvis. His white blood cell count was 8.2 × 10^3^/*μ*L, hemoglobin 14.8 g/dL, hematocrit 45%, and platelet count 219 × 10^3^/*μ*L. The differential count in the peripheral blood was 34% neutrophils, 2% bands, 57% lymphocytes, 5% monocytes, and 2% eosinophils.

**Figure 1:**
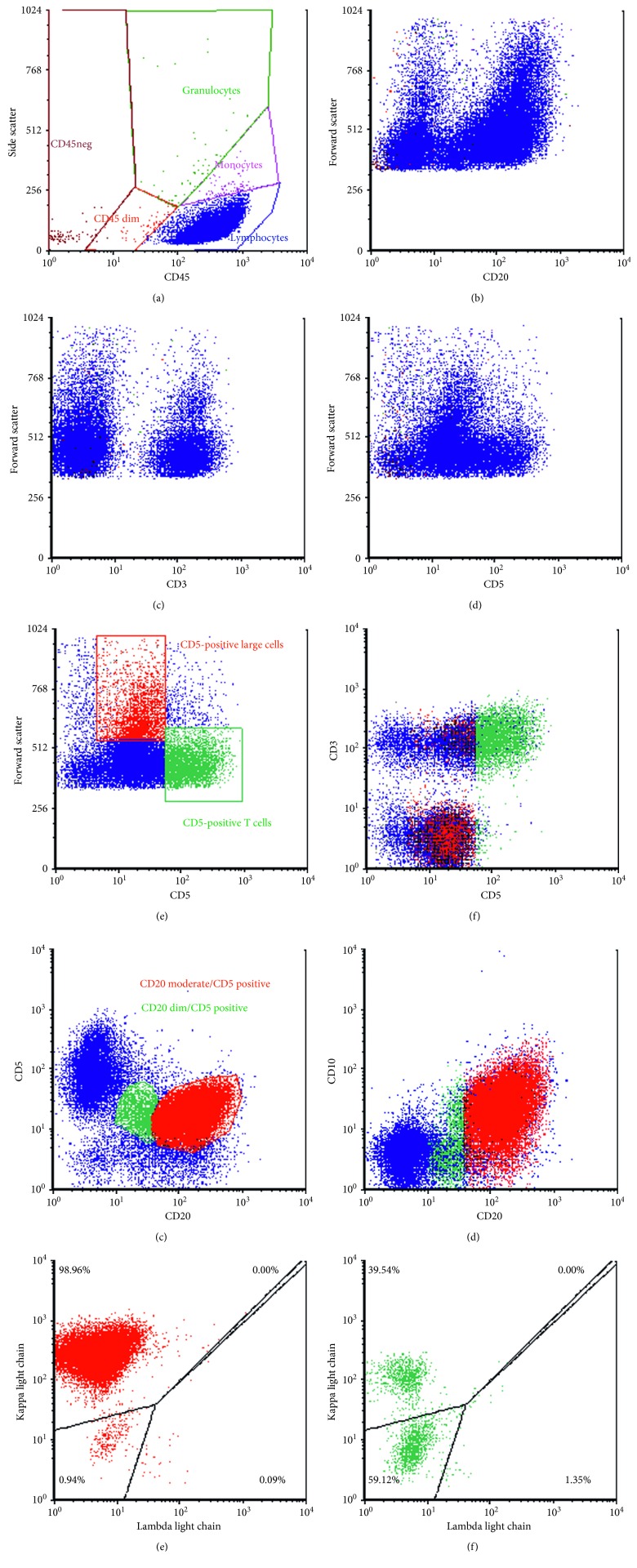
Flow cytometry on the lymph node. (a) The majority of cells express bright CD45 with low side angle light scatter characteristic of lymphoid cells. (b) B-cells expressing CD20 range from small to large in size by forward light scatter. (c, d) The large neoplastic cells are positive for CD5 and negative for surface CD3. Cells showing the brightest intensity for CD3 and CD5 are background T-cells. (e, f) Large cells gated in red express dim CD5, but not surface CD3. The cells staining brightest for CD5 and gated in green are background T-cells and also stain for CD3. (g, h) Most CD20 moderate B-cells coexpressing CD5 gated in red also express CD10, while most CD20 dim B-cells gated in green do not express CD10. (i) Most of the CD20 moderate B-cells in red show moderate surface expression of kappa light chain. (j) Most of the CD20 dim B-cells in green do not show any surface light chain expression.

An excisional biopsy of the left inguinal mass was performed. Flow cytometric phenotyping identified the presence of two distinct, phenotypically abnormal B-cell populations (Figure 1). The minority population was composed of small cells expressing moderate CD19, dim CD20, moderate CD5, dim CD11c, and dim CD23 with no definitive surface light chain expression. The majority population was composed of medium- to large-sized cells expressing moderate CD19, moderate CD20, dim to moderate CD5, and moderate CD10 along with a bright surface kappa light chain restriction. This majority population did not express CD11c or CD23.

**Figure 2:**
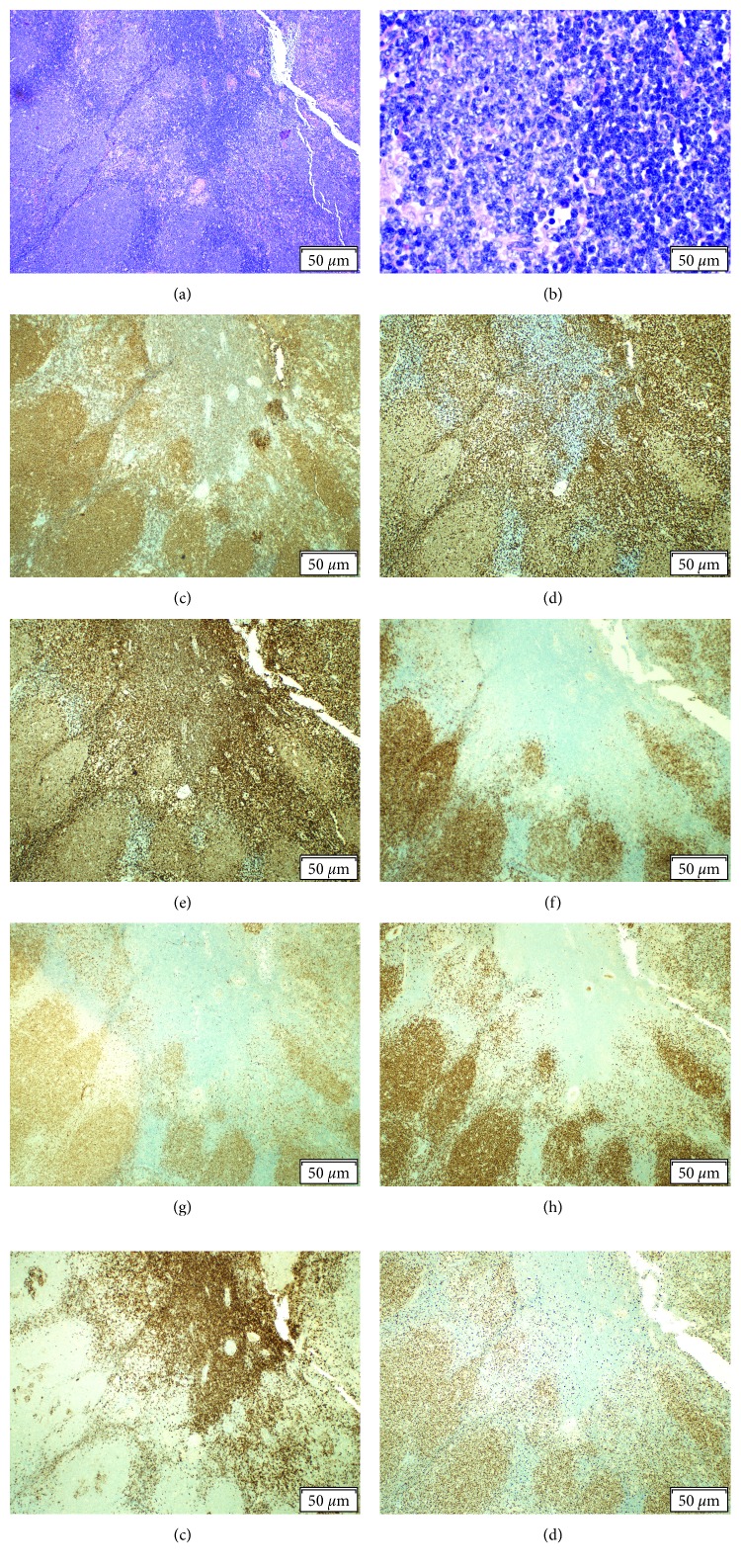
(a) Hematoxylin and eosin stain 40x: the lighter staining nodular areas contain the larger cells of the blastoid variant mantle cell lymphoma (MCL). The darker staining areas near the center contain the small cells of the small lymphocytic lymphoma (SLL) component. (b) Hematoxylin and eosin stain 400x: the larger cells of the MCL are on the left and smaller cells of the SLL are on the right. (c) The MCL and SLL both stain for CD20. The SLL displays weaker staining. (d) The large nodules of the MCL stain weakly for CD3. The SLL is negative. (e) Both MCL and SLL stain for CD5. (f) Only the nodules of MCL stain for CD10. The SLL is negative. (g, h) The MCL stains for CyclinD1 (BCL-1) and SOX-11. The SLL component is negative. (i) The SLL in the center stains for CD23. (j) Many cells in the MCL nodules stain with Ki-67 indicating a high proliferation rate, whereas very few stain in the center SLL region stain indicating a low proliferation rate.

Morphologic review of hematoxylin and eosin (H&E) stained fixed tissue sections and immunohistochemistry (IHC) confirmed the presence of two B-cell populations (Figure 2). Expanses of large lymphoid cells with vesicular chromatin and nucleoli stained positive for CD20, CD3, CD5, CD10, CyclinD1 (BCL-1), and SOX-11 with a high estimated proliferation rate (Ki-67) of 70%. A second population of small B-cells between the larger cells stained less intense for CD20, coexpressing CD5 and CD23. These smaller cells were negative for CD3, CD10, CyclinD1 (BCL-1), and SOX-11 with a low estimated proliferation rate (Ki-67) of <10%.

**Figure 3:**
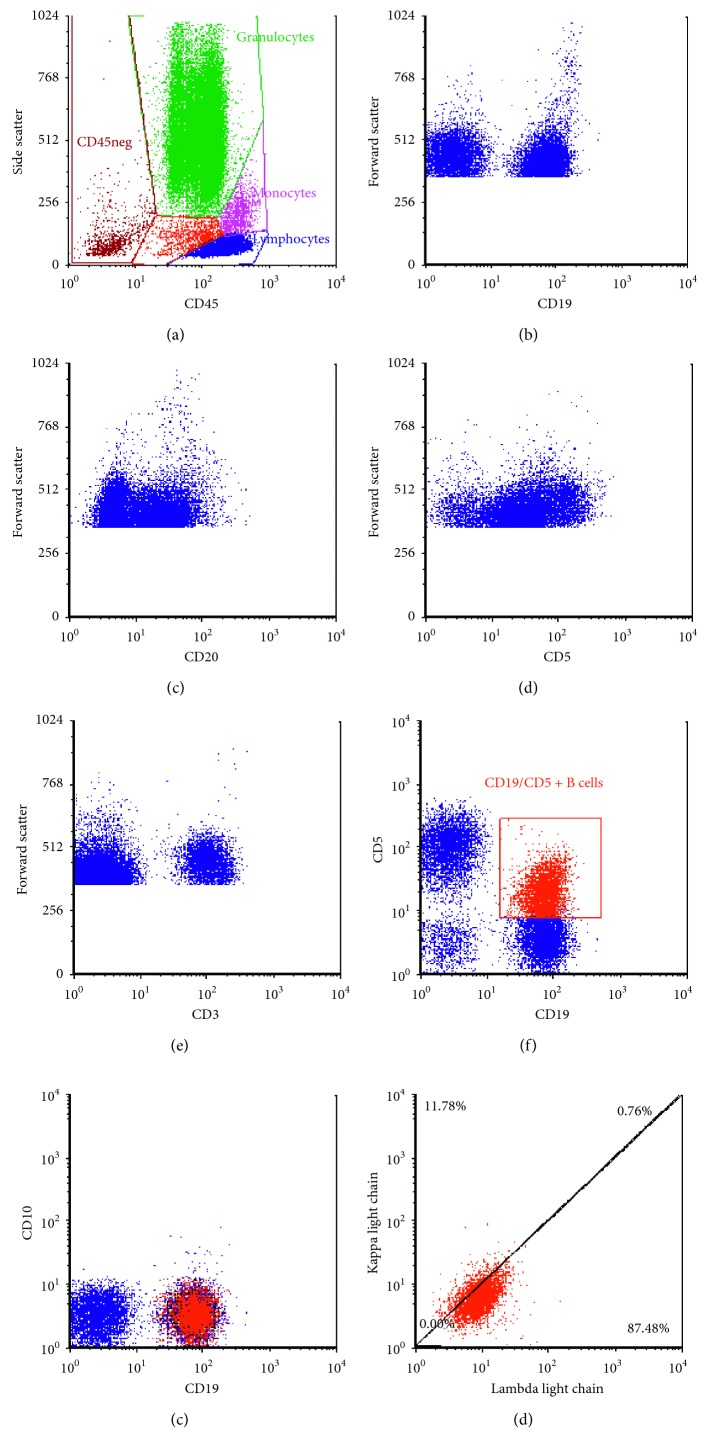
Flow cytometry on the bone marrow. (a) The following images come from the region gated as “lymphocytes”. (b, c) The B-cells are small in size by forward light scatter and express moderate intensity CD19 and dim intensity CD20, a feature typical of chronic lymphocytic lymphoma/small lymphocytic lymphoma (CLL/SLL). This differs from the large size and intensity of these antigens in the mantle cell lymphoma of the lymph node. (d, e) The small B-cells stain for CD5 but are negative for CD3. The cells expressing bright CD3 are the T-cells. (f) A region color coded with orange represents the B-cells coexpressing CD5. (g, h) These cells do not express CD10 and the surface light chain expression is negative or equivocal.

Flow cytometric phenotyping of the staging bone marrow aspirate detected small B-cells with features most often associated with chronic lymphocytic leukemia/small lymphocytic lymphoma (CLL/SLL) (Figure 3). These features included small B-cells coexpressing moderate intensity CD19, dim CD20, dim CD5, dim CD11c, dim CD23, and no definitive surface light chain expression. These B-cells did not express CD3, CD10, or FMC-7. Morphologically, there were multiple small aggregates in the core and clot sections composed of B-cells staining for CD5 without CD3, CD10, or CyclinD1 (BCL-1).

Microdissection of the lymph node into MCL and SLL components based on an H&E stain was performed in order to perform additional FISH testing for IgH/CCND1 and B- and T-cell gene rearrangement studies. B-cell gene rearrangements for 3 framework regions of the heavy chain (FR-JH), VK-JK, and VK-KDE light chain and T-cell rearrangements for VB-JB1-DB1, VB-JB2, V1-8-J1.3–2.3, V9-J1.3–2.3, and V10-J1.3–2.3 were performed using polymerase chain reaction (PCR). The same molecular studies were performed on the aspirate clot section of the bone marrow (Tables 1 and 2).

B-cell gene rearrangement studies on the bone marrow aspirate clot (SLL only) revealed the following prominent peaks: 2 peaks for FR2-JH, 2 peaks for FR3-JH, 2 peaks for DH-JH, 1 peak for DH7-JH, 2 peaks for VK-JK, and 1 peak for VK-KDE. All of these clonal peaks were also seen in the MCL component of the lymph node. There was an additional prominent peak for FR1-JH, 2 additional peaks for DH-JH, and 2 for VK-JK in the MCL component of the lymph node. T-cell gene rearrangements were detected in both the bone marrow aspirate clot and the MCL component of the lymph node. Two identical clonal peaks for VB-JB2-Db2 and one identical peak for VB-JB1-Db1 were detected in the bone marrow aspirate and MCL component of the lymph node. Both the bone marrow aspirate clot and the MCL component of the lymph node showed additional, separate prominent peaks for other T-cell primer sets.

Fluorescence in situ hybridization (FISH) using dual-fusion probes confirmed the presence of the t(11;14)(q13;q32)–IgH/CCND1 translocation consistent with MCL in the lymph node. The bone marrow aspirate clot was negative.

Based on all of the results, the diagnosis of lymph node involvement by clonally related blastoid variant mantle cell lymphoma with aberrant CD10 and CD3 and small lymphocytic lymphoma was made. The staging marrow was involved by SLL, but not by MCL.

## 3. Discussion

The SLL component in the lymph node and bone marrow demonstrated typical morphologic and phenotypic features. This included small lymphoid cells with condensed chromatin that expressed moderate intensity CD19, dim intensity CD20, CD5, CD11c, and CD23 with negative or equivocal intensity surface light chain and no evidence of FMC-7. These cells had a low estimated proliferation rate by Ki-67 evaluation in the lymph node (<10%) and did not stain for CyclinD1 (BCL-1). In contrast, the large size cell component effacing most of the lymph node, and not detected in the bone marrow, showed a very uncommon immunophenotype. In addition to the expected expression of CD20 and CD5, it also expressed CD10, which is typically considered a marker for follicular center cell derivation, and it expressed cytoplasmic CD3, which is a T-cell antigen. FISH detected an IgH/CCND1 translocation in the lymph node, but not in the bone marrow aspirate clot. Polymerase chain reaction (PCR) demonstrated B-cell heavy and light chain gene rearrangements and T-cell receptor gene rearrangements in both the lymph node and bone marrow aspirate clot.

In the lymph node, these studies raise the differential diagnosis of blastoid variant MCL, diffuse large B-cell lymphoma germinal center cell type (DLBCL), Richter's transformation of the SLL, and T-cell lymphoma (TCL).

CD20 expression has been reported in TCL [8–11]. However, none of the TCLs reported in the literature expressed surface light chains. Most TCLs also stained for other T-cell markers in addition to CD3, whereas this case did not show expression of CD2, CD4, CD7, or CD8 by flow cytometry. The CD3 was detected by immunohistochemistry and not by flow cytometry, suggesting that the malignant cells contained cytoplasmic but not surface CD3. Studies of normal T-cell development and cases of T-cell acute lymphoblastic leukemia indicate that immature T-cells contain cytoplasmic CD3, T-cells of intermediate differentiation will express both cytoplasmic and surface CD3, and the most mature T-cells express surface CD3 [12]. So, expression of a cytoplasmic without surface CD3 T-cell receptor is possible and can suggest a T-cell lineage.

Cases of B-cell lymphoma expressing CD3 have been reported. A case of CyclinD1-negative mantle cell lymphoma recently published showed expression of cytoplasmic CD3 epsilon by immunohistochemistry [13]. That case did not show a TCR gene rearrangement. A series of 21 cases of B-cell lymphoma, including 12 diffuse large B-cell lymphomas (DLBCL), 2 plasmablastic lymphomas, 4 plasma cell neoplasms, 2 Burkitt lymphomas, and 1 grade 3A follicular lymphoma, expressed CD3 [14]. Of these cases, DLBCL with plasmacytic differentiation, plasma cell neoplasms, and plasmablastic lymphomas showed cytoplasmic localization of the CD3 without surface expression. None of the cases in that study demonstrated a clonal TCR gene rearrangement.

Of interest is a report of primary mediastinal large B-cell lymphoma showing CD3 expression with dual clonal immunoglobulin (Ig) and TCR gene rearrangements [15]. That article also referenced two additional cases of B-cell lymphoma with both Ig and TCR gene rearrangements. Of the 14 total cases reviewed in that article, 11 of the cases tested positive for EBV, which could play a role in lineage infidelity. However, due to the limited number of cases available, the cause or prognosis of TCR gene rearrangement and cytoplasmic CD3 expression in B-cell lymphomas is still otherwise unknown.

In this case, the coexpression of CD19, CD20, and surface light chain with only cytoplasmic CD3 strongly supports a B-cell rather than T-cell lineage despite the presence of a clonal TCR gene rearrangement. The expression of CyclinD1 (BCL-1) detected by immunohistochemistry and t(11;14)(q13;q32)–IgH/CCND1 translocation detected by FISH further supports MCL. The larger cell size, more open chromatin, and high estimated proliferation rate by Ki-67 are findings consistent with the blastoid variant. CD10 expression is not commonplace in MCL but has been reported [16, 17]. One study of 17 specimens from 13 patients detected CD10 expression in 14 of the samples [18]. Five of those showed pleomorphic blastoid morphology. In that study, sequence analysis of the Ig heavy chain variable region in 5 of the cases showed only 1 to have low-level somatic mutation, indicating they did not arise from germinal center cells. However, a separate series of 9 cases of CD10-positive MCL showed gene expression profiles with a germinal center cell derived signature but no significant differences in the clinicopathological features or clinical outcome between the CD10-positive and CD10-negative cases [19].

We investigated the clonal relationship between the MCL and SLL components using B-cell immunoglobulin heavy chain (IgH) and kappa light chain and T-cell gene rearrangement studies for beta and gamma T-cell receptor (TCR) by PCR to compare fragment lengths of clonal peaks. Unfortunately, deeper sectioning into the fixed tissue paraffin block for previous studies had severely reduced the SLL component, making separation between the MCL and SLL components extremely difficult in the lymph node. We suspect there was cross-contamination of MCL into the SLL areas sampled. This was supported by the FISH for IgH/CCND1 being positive at near 70% in both dissection areas despite negative CyclinD1 (BCL-1) and SOX-11 IHC and absence of IgH/CCND1 by FISH in the bone marrow aspirate clot. According to all morphologic, immunophenotypic, and FISH studies, the bone marrow aspirate clot harbored only the SLL component without the MCL component. Therefore, the comparison was made between the gene rearrangement studies from the bone marrow aspirate clot with SLL only and the MCL component of the lymph node. All of the clonal B-cell gene rearrangement peaks in the bone marrow aspirate clot were also found in the MCL component of the lymph node with the same base pair peak sizes. This strongly supports a clonal relationship between the SLL in the bone marrow and the MCL in the lymph node. There were numerous additional, unique B-cell gene rearrangement peaks only in the lymph node, including 1 additional peak for FR1-JH, 2 additional peaks for DH-JH, and 2 additional peaks for VK-JK-a primers. There were also 3 common clonal peaks of like size between the bone marrow aspirate clot and the MCL component of the lymph node for T-cell gene primers. Several additional separate TCR peaks of different sizes were only detected either in the bone marrow aspirate clot or in the lymph node.

In a published series of eleven composite MCL and SLL cases, there was no convincing clonal link between the two components following microdissection and B-cell gene rearrangement studies by PCR [20]. Interestingly, all the cases in that study also showed multiple clonal peaks of similar fragment size in both the MCL and SLL components. The authors reasoned that biclonal (biallelic) rearrangements in one component, especially the SLL, of the composite cases were very unprobable and that the results were better interpreted to indicate that, despite microdissection, isolated tumor cells particularly of the MCL component had “contaminated” the specimens.

A different study investigating the clonal relationship between 27 primary and recurrent B-cell lymphomas showed one case where the PCR fragment lengths were off by 3 base pairs, but gene sequencing revealed identical heavy chain V, D, and J gene segments and junctions [21]. Another case in that series detected clonal peaks off by only a single base pair, yet gene sequencing revealed different V, D, and J gene segments. In 17 cases analyzed by gene sequencing, an identical VDJ gene rearrangement was confirmed in 4/4 primary and recurrent lymphoma pairs with the same fragment lengths by PCR and in 10/13 pairs (77%) with different fragment lengths by PCR. Based on these results, they concluded that clonal relationships determined by sequencing of the immunoglobulin genes were more reliable than by comparing the PCR product size alone. Our case showed 9 clonal peaks of the same fragment lengths for B-cell heavy and light chain gene rearrangement studies and 3 clonal peaks of the same fragment lengths for T-cell gene rearrangement studies between the pure SLL component from the marrow and the MCL population in the lymph node using multiple tubes with different primer sets. We feel this strongly supports a clonal relationship between the SLL and MCL populations without the need for direct sequencing.

Another interesting case report described detailed molecular-cytogenetic and backtracking analysis of a patient diagnosed with chronic lymphocytic leukemia (CLL) in the blood which progressed, prompting treatment with fludaribline, cyclophosphamide, and rituximab followed by an autologous stem cell therapy [22]. Following a remission of 3 years, the patient developed a gradually increasing lymphocytosis over the course of another 3 years that progressed to stage IV disease with advanced lymphadenopathy, splenomegaly, and thrombocytopenia. A biopsy of a cervical lymph node revealed CyclinD1-positive MCL. The bone marrow at relapse showed evidence of both CLL and MCL. PCR analysis of the unique IgHV gene sequences confirmed that the MCL and CLL population were clonally related, likely through a common clonal progenitor. The authors designed a qPCR assay using mutation-specific primers spanning the site for the focal deletion in TP53 found in the MCL clone but not in the CLL clone. They also used a separate qPCR assay detecting the clonal IgH rearrangement found in both the CLL and MCL clones. They tested a series of archived samples from blood and bone marrow collected from the patient over a period of 9 years using these primer sets. This backtracking analysis revealed that the patient never achieved complete remission from the CLL and that the pre-MCL clone with a focal TP53 deletion appeared in the BM and PB of the patient approximately 4 years before the clinical manifestation of MCL. Whole genome sequencing of CLL cells obtained from the peripheral blood of the patient before initiation of second line therapy for the first relapse of CLL and the MCL cells collected during the later appearance of MCL demonstrated several mutations in prognostically relevant genes acquired by the pre-MCL and MCL clones. The authors concluded that molecular and cytogenetic backtracking data confirmed a slow development of a pre-MCL clone, which with high probability originated from a minor CLL clone.

Herein we present a diagnostic challenge of MCL due to the presence of a concurrent SLL in addition to aberrant coexpression of cytoplasmic CD3 and surface CD10 along with documented TCR receptor gene rearrangements. Keys to the diagnosis included the identification of morphologically and phenotypically distinct populations using flow cytometric and immunostain phenotyping. Despite the distraction of cytoplasmic CD3 and surface CD10 expression, the diagnosis of MCL was confirmed by the classical coexpression of CD19, CD20, CD5, CyclinD1 (BCL-1), SOX-11, and surface light chain together with documentation of an IgH/CCND1 translocation by FISH. In addition, B- and T-cell gene rearrangement studies by PCR supported a clonal relationship between the SLL and MCL components. As an entity, MCL is aggressive and the blastoid subtype has a poor prognosis [23]. In one study of 187 patients, 33 (17%) were diagnosed with MCL [24]. The median overall survival time was 14.5 months for the 33 blastoid variant MCL patients as compared to 53 months for the 154 patients with the more common form of MCL. Recognition of the entity and differentiation from other more indolent forms of B-cell lymphoma and T-cell lymphoma is important for proper therapy and prognosis.

## Figures and Tables

**Table 1:** Comparison of clonal peaks for B-cell gene rearrangements from the bone marrow aspirate clot (SLL) and lymph node (MCL) detected by polymerase chain reaction (PCR).

Clonal peaks—B-cell gene rearrangement by PCR		
Primer region	Bone marrow—SLL (fragment size/peak height)	Lymph node—MCL (fragment size/peak height)
FR1-JH		*348.71 (32369)*
FR2-JH	**259.94 (3348)**	**259 (small)**
	**279.93 (3173)**	**283.13 (32055)**
FR3-JH	**116.96 (3225)**	**116 (tiny)**
	**∼149 (∼1500)**	**149.33 (10235)**
DH-JH-a		*245.16 (10839)*
		*∼254 (>10000)*
	**∼345**	**∼345**
	**393.00 (1506)**	**393 (tiny)**
DH7-JH	**115 (tiny)**	**115.70 (1702)**
VK-JK-a		*143.78 (>10000)*
	**145.08 (32701)**	**∼145 (>10000)**
		*∼149 (>10000)*
	**197 (tiny)**	**197.20 (1050)**
VK-KDE	**284.84 (1803)**	**Oligoclonal**

*Note.* Peaks of the same fragment length are in bold. Peaks present in only one component are in italics.

**Table 2:** Comparison of clonal peaks for T-cell gene rearrangements from the bone marrow aspirate clot (SLL) and lymph node (MCL) detected by polymerase chain reaction (PCR).

Clonal peaks—T-cell gene rearrangement by PCR		
Primer region	Bone marrow—SLL (fragment size/peak height)	Lymph node—MCL (fragment size/peak height)
VB-JB2	*255.55 (3857)*	
VB-JB1	Polyclonal	Polyclonal
VB-JB2-Db2	**182.43 (911)**	**182.26 (11656)**
	**191.66 (1772)**	**∼191 (∼5000)**
VB-JB2-DB1	*312.00 (1837)*	
VB-JB1-Db1	**297.79 (1817)**	**297.73 (2640)**
V10-J1.3–2.3	*161.23 (3630)*	
		*152.85 (4576)*
V1-8-J1.3–2.3	*203.87 (2622)*	
		*216.04 (5578)*
V9-J1.3–2.3		*174.29 (6122)*

*Note.* Peaks of the same fragment length are in bold. Peaks present in only one component are in italics.
